# An assessment of current concussion identification and diagnosis methods in sports settings: a systematic review

**DOI:** 10.1186/s13102-022-00514-1

**Published:** 2022-07-10

**Authors:** Ed Daly, Alan J. Pearce, Emma Finnegan, Ciara Cooney, Maria McDonagh, Grainne Scully, Michael McCann, Rónán Doherty, Adam White, Simon Phelan, Nathan Howarth, Lisa Ryan

**Affiliations:** 1 Department of Sport, Exercise and Nutrition, School of Science and Computing, Atlantic Technological University, Galway, Ireland; 2 College of Sport, Health and Engineering, La Trobe University, Melbourne, Australia; 3 Atlantic Technological University, Port Road, Letterkenny, Ireland; 4 Faculty of Health and Life Sciences, Oxford Brookes University, Oxford, England

**Keywords:** Concussion, Head injury assessment, Screening tools, Assessment protocols, Sport injury risk, SCAT

## Abstract

**Background:**

Concussion in sport is an ongoing global concern. The head injury assessment (HIA) by the field of play is acknowledged as the first step in recognising and identifying concussion. While previous systematic literature reviews have evaluated the sensitivity of side-line screening tools and assessment protocols, no systematic review has evaluated the research designs and assessments used in a field setting. This systematic review investigated existing screening and diagnostic tools used in research as part of the HIA protocol to identify concussion that are currently used in professional, semi-professional and amateur (club) sports settings.

**Methods:**

A systematic searching of relevant databases was undertaken for peer-reviewed literature between 2015 and 2020.

**Results:**

Twenty-six studies met the inclusion criteria. Studies were of moderate to good quality, reporting a variety of designs. The majority of studies were undertaken in professional/elite environments with medical doctors and allied health practitioners (e.g., physical therapists) involved in 88% of concussion assessments. While gender was reported in 24 of the 26 studies, the majority of participants were male (77%). There was also a variety of concussion assessments (n = 20) with the sports concussion assessment tool (SCAT) used in less than half of the included studies.

**Conclusion:**

The majority of studies investigating concussion HIAs are focused on professional/elite sport. With concussion an issue at all levels of sport, future research should be directed at non-elite sport. Further, for research purposes, the SCAT assessment should also be used more widely to allow for consistency across studies.

**Supplementary Information:**

The online version contains supplementary material available at 10.1186/s13102-022-00514-1.

## Key points

- Head injury assessments are vital as part of the diagnostic pathway for suspected concussion at all levels of sport.
- This systematic review found that in studies between 2015 and 2020, the majority were focused on elite sports and male participants.
- Studies also utilized a disparate array of assessments making comparison difficult.
- Future studies should aim to focus on amateur (club) sports, include more female participants, and as a minimum include the sports concussion assessment tool (SCAT), along with other assessments to allow for consistency across studies.

## Background

Despite consensus on the clinical definition of concussion in sport, its immediate and accurate recognition in a clinical setting/pitch-side setting remains a challenge to implement beyond the elite level of sport. Currently, only a licensed medical practitioner is entitled to diagnose a concussion [1]. However, even at the elite level, clinical decision-making is guided on subjective athlete self-report with observation of symptoms and severity [1]. Further, determination of when an athlete is appropriately ready to return to sport is limited also by subjective symptom scores and imperfect clinical and neuropsychological testing [2]. Despite these challenges, the consensus remains a medical decision based upon resolution of symptoms, as reported by the athlete, as well as completing the return to sport guideline protocols [1].

It is important to accurately diagnose a concussion and have the athlete complete a full recovery. It is well described that sustaining a concussion increases the likelihood of incurring a subsequent injury of approximately two-fold [3, 4]. While epidemiological studies only describe the risk, observational studies have suggested that increased risk appears to be due to continuing neurological and neuromuscular impairments post-concussion [5–7]. It has been suggested that experiencing numerous concussions, or exposure to repetitive sub-concussive head trauma, could be associated with long-term consequences such as persistent post-concussive symptoms [8] or increasing risk of neurodegenerative disorders such as chronic traumatic encephalopathy, Alzheimer’s disease, Parkinson’s disease and motor neuron disease [9–13].

Given these risks, it is important that athletes suspected of concussion following an impact are assessed and identified via the removal of participants for further evaluation and, if diagnosed with concussion, consequently engaged in a graduated return to play protocol [1]. At the elite levels of competitions, the use of head injury assessment (HIA) tools, such as the Sports Concussion Assessment Tool (SCAT) have been evolving since the first consensus statement published in 2001 [14]. While these assessment tools do not replace the medical decision, their use certainly assists the medical practitioner with clinical decision making as well as guidance for return to play clinical decisions for athletes post-concussion. Previous systematic literature reviews have evaluated the sensitivity of side-line screening tools and assessment protocols [15–17]. To date no systematic review has evaluated the overall efficacy of these tools and protocols in a field setting. Moreover, it is unknown if HIAs are utilized in non-elite environments, such as amateur club competitions. This systematic review and qualitative analysis aimed to investigate the prevalence and type of current off-field or ‘side-line’ recognition of suspected concussions. The primary objective was to investigate existing screening and diagnostic tools that are used in identifying concussion, or head injury assessment protocols that are currently used in professional, semi-professional and amateur (club) sports settings.

## Methods

### Study design

The review protocol was prospectively registered in the PROSPERO database for systematic reviews (protocol ID: CRD42021214339) and complies with Preferred Reporting Items for Systematic Reviews and Meta-analyses (PRISMA) guidelines [18] (Additional file 2). The review question and inclusion/exclusion criteria and search terms are presented in Table 1.

**Table 1:** PICO-model and medline search strategy in accordance with PRISMA statement

Primary review question/aim	
What are the current side-line screening methods used to establish the diagnosis of acute concussion or suspected concussion across sports in an adult population?	
*Inclusion criteria*	
Population	Athletes aged 18 years or greater, involved in amateur, semi-professional or professional sport and sustaining a suspected concussive injury. For the purpose of this review, we allowed for the inclusion of athletes younger than 18 years (e.g., studies with an age range of 5–23 years). These studies were considered important as the cohort included were over 18 years of age
Intervention	Any side-line* screening assessment used to detect suspected concussion following sports-related head impact event in the acute phase of injury. For this review the acute phase of injury will be defined as minutes after the event up to and including 7 days post event. These will include (but may not be limited to) reported (i). Concussion 2, (ii) mTBI, (iii) Cervical neck injury
Outcomes	Acute concussion diagnosis methods
Study design	Published research, retrospective data analysis, cross sectional study design, parallel studies, prospective, observational, systematic reviews where data meeting the PICO can be extracted. Abstracts (with data) will be included initially. Research published from 2015 onwards
*Exclusion criteria*	
Population	Not related to sport, animal studies or studies in which all participants were under 18 years of age
Intervention	Non-side-line testing, testing conducted > 7 days post event
Outcomes	Concussion/suspected concussion not examined side-line or diagnosis assessment requiring advanced medical training/technology or referral to secondary care for diagnosis to be made
Study design	Case reports, editorials, commentary’s, review articles (in the case of systematic reviews if relevant data cannot be extracted or does not meet PICO), consensus statements, position stands and non-English publications. Research published in 2014 and prior
*Search terms*	
^†^((sport related concussion OR SRC OR mTBI OR mild traumatic brain injury) AND (diagnosis OR treatment)) AND (sport)	

*‘Side-line’ is used generally to denote testing away from the immediate sporting environment, for example rink side, track-side, locker room, medical room, touch line and so on. ^†^MeSH terms were exploded to include more specific terms; MeSH terms were translated into the appropriate subject headings for other databases. Keywords were the same for each database searched

### Identification of evidence

An electronic search was conducted on 01 July 2020. The following electronic databases were queried: PsychInfo (OVID), PubMed, Science Direct, SPORTDiscus (EBSCOhost), Web of Science, and the Cumulative Index to Nursing and Allied Health Literature (CINAHL) (EBSCOhost). The search terms included: (sport related concussion OR SRC OR mTBI OR mild traumatic brain injury) AND (diagnosis OR treatment) AND (sport). Articles met inclusion (eligibility criteria) based on the following priori inclusion criteria: (i) participants were involved in professional/elite, semi-professional/sub-elite or amateur (club) sport at the time of injury; (ii) individuals aged 18 years or greater; (iii) concussion diagnosis assessment was administered acutely (here, we define acute as ≤ 7 days of injury); (iv) peer-reviewed journal articles published since 2015. Articles prior to 2015 would not be reflective of concussion recognition strategies as outlined in the most recent Concussion Consensus Statement (2016). However, part of our *a-priori* inclusion criteria was modified. For the purpose of this review we allowed for the inclusion of athletes younger than age 18 years (e.g., studies with an age range of 5–23 years). These studies were considered important as a majority of athletes in these included studies were 18 years or older. Studies in which all participants were under 18 years of age were not included. Full detail on the search strategy and inclusion and exclusion criteria are reported in Table 1.

### Selection of evidence and data extraction

**Fig. 1:**
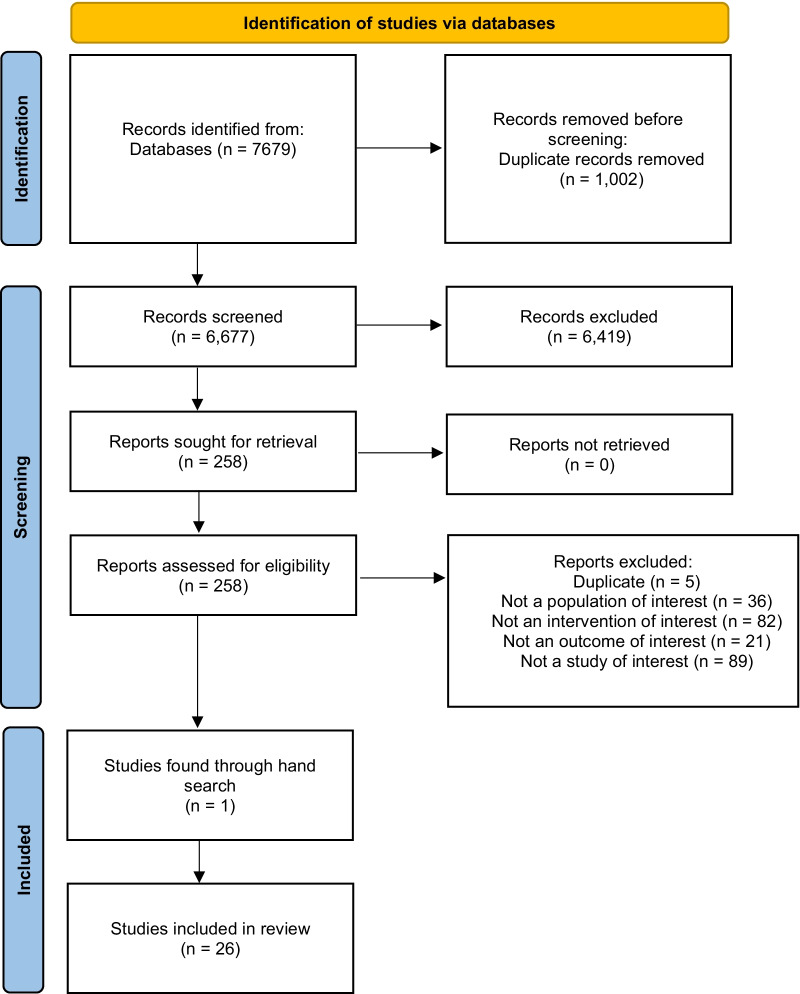
Preferred reporting items for systematic reviews and meta-analysis flow diagram of the studies included in the review [18]

Following compilation of online search results, record titles and abstracts were screened by four authors (CC, ED, MMD, AJP), full text articles were reviewed by five authors (CC, ED, MMD, AJP, LR). The reference lists from review articles were assessed for pertinent studies that may have been overlooked. Data extraction was performed independently by two authors (GS and ED) and data reviewed by a third author (CC) for consistency and accuracy. In cases of disagreement at any stage, consultations with other authors (CC, ED, MMD, AJP, LR) were planned and disagreement resolved by joint discussion and consensus. Figure 1 illustrates the literature review process using PRISMA flow chart [18].

### Data synthesis, statistical analyses and assessment of overall quality of evidence

References were managed in EndNote (Clarivate Analytics, Berkeley California, USA), extracted data were collated in Excel 2013. The overall quality of evidence for each outcome was assessed using a modified Downs and Black [19] checklist for measuring study quality by three authors (AP, ED, LR). Higher total scores for this checklist reflect increased study quality and confidence in conclusions, but we used the stratification of poor (< 7), moderate (8–15), good (> 16)[20]. However, as some questions were not pertinent to this review, a modified form of the checklist was used for a maximum score of 22. Due to the heterogeneity of the studies a meta-analysis could not be performed, and a qualitative analysis of studies was instead conducted.

## Results

### Study selection

A total of 7679 citations were screened for eligibility, with the full text of 258 articles retrieved for detailed evaluation. Twenty-six studies met the inclusion criteria following full text review. Figure 1 presents the PRISMA flow diagram [18] of identification, screening, eligibility and inclusion criteria for the literature review of side-line diagnosis of concussion. Table 2 presents the studies meeting the inclusion criteria.

**Table 2:** Overall study characteristics, level of evidence [44], and modified Downs and Black [19] checklist (maximum score 22)

Author (year)	Study type	Level of evidence	Assessment	Sample size (concussed/control)	Sex M/F	Mean age (range) years	Sport	Test post-concussion	Score (max 22)
Broglio et al. [25]	PCS	III-2	BESS, SAC, SCAT5, ImPACT, VOMS, BSI-18	N = 1458	919/539	19.0 (N/A)	NCAA (sport unspecified)	3 times in 72 h (h): 0–1.25 h (side-line),1.25–24 h (post-event), 24–72 h (clinic)	17
Buckley et al. [45]	Cohort	III-2	GI	N = 84 (42/42)	40/44	19.2 (N/A)	NCAA (sport unspecified)	24 h	16
Downey et al. [21]	PCS	III-2	SCAT3	N = 45 (23/22)	19/26	20.0 (N/A)	Football, rugby, ice hockey, soccer, lacrosse, basketball, volleyball, field hockey, baseball, wrestling	3 to 5 days (acute), 3 weeks (post-acute)	14
Fallon et al. [30]	PCS	III-2	MULES, SCAT3	N = 681 (17)	422/259	17 (6–37)	Ice hockey, soccer, football	Side-line	13
Fuller et al. [31]	Cross Sectional	III-2	SCAT3	N = 639 (24)	All male	27.4 (N/A)	Rugby	After game of injury	14
Fuller et al. [46]	Pilot	III-3	PSCA1	N = 165	All male	N/A	Rugby	Side-line (Time frame not stated)	17
Fuller et al. [37]	PCS	III-3	KD	N = 261	All male	27.6 (N/A)	Rugby	48 h	18
Fuller et al. [38]	RCS	III-2	HIA01/ SCAT3	N = 1265	N/A	N/A	Rugby	Side-line	16
Galetta et al. [26]	PCS	III-2	KD, SAC, TG	N = 332	192/140	11.0 (5–23)	Ice hockey, lacrosse	Side-line /rink-side. (Time frame not stated)	15
Gardner et al. [39]	Observational	IV	VA	N = 400	All male	N/A	Rugby league	N/A	
Goble et al. [47]	Cross-Sectional	III-2	BBT	N = 25	11/14	20.7 (N/A)	College athletes- (unspecified)	48 h	9
Graves [28]	PCS	III-2	SOT, BESS	N = 15 (15)	All male	18.9 (N/A)	Football	1–14 days	15
Hänninen et al. [32]	PCS	III-3	SCAT3	N = 283 (27)	All male	27.0 (N/A)	Ice hockey	24 h	15
Harrold et al. [33]	PCS	III-3	KD, SCAT3	N = 426	177/249	35.0 (N/A)	Sport, other	N/A	16
Hecimovich et al. [36]	PCS	III-2	KD	N = 22 (7/15)	All male	19.6 (N/A)	Australian football	10–20 min post-game	14
King et al. [34]	PCS Observational	III-2	KD, SCAT3	N = 104 (52/52)	All male	23.7 (N/A)	Rugby	Days 3, 7, 14, and 21	15
Leong et al. [48]	PCS	III-3	KD, SCAT2	N = 127 (11)	119/8	19.5 (N/A)	Football, basketball	N/A	17
Marinides et al. [27]	RCS	III-3	KD, PCS, SAC, BESS, ImPACT	N = 221 (30)	150/71	N/A	Football, lacrosse, soccer	87 min	14
Merritt et al. [49]	PCS	III-2	PCSS, ImPACT	N = 846 (86)	637/209	19.9 (N/A)	Football, basketball, ice hockey, soccer, lacrosse, wrestling, other	2, 7, and 14 days post-injury	18
Molloy et al. [50]	Descriptive cohort	III-2	KD, PSCA2, CogSport	N = 176 (19/33)	All male	23.8 (N/A)	Rugby	48 h	18
Oldham et al. [22]	Prospective, longitudinal	III-1	TG, BESS, mBESS	N = 76 (38/38)	All male	N/A	NCAA student-athletes	< 48 h	16
Putukian et al. [51]	Prospective cross-sectional	III-1	SCAT2	N = 263 (85/178)	184/79	20.3 (N/A)	Football, rugby, volleyball, football, crew	0.52 ± 1.18 days	13
Russell-Giller et al. [29]	Pilot	IV	KD, VOMS	N = 71	N/A	14.0 (N/A)	Sports (unspecified), other	1–5 days	17
Seidman et al. [35]	PCS	III-3	KD, SCAT3	N = 337 (9/328)	All male	15.0 (N/A)	American Football	72 h	12
Sufrinko et al. [23]	Case–control	III-2	ImPACT, SAC, BESS	N = 125 (125)	85/40	16.8 (N/A)	Football, soccer, volleyball, basketball, wrestling, ice hockey, softball,	SAC, BESS: 24 to 48 h, ImPACT: 5 to 7 and 10 to 14	13
Vartiainen et al. [24]	PCS	III-2	SCAT3, MotCoTe	N = 16 (9/7)	All male	23.4 (N/A)	Ice hockey	36 h	15

*N/A* Not available, *KD* King Devick test, *GI* Gait Initiation, *SOT* Sensory Organization, *TG* Tandem Gait, *GT* Gait Termination, *MULES* Mobile Universal Lexicon Evaluation Systems, *HIA01* Head Injury Assessment Version 1, *PSCA1* Pitch-Side Concussion Assessment Version 1, *PSCA2* Pitch-Side Concussion Assessment Version 2, *MotCoTe* Motor Cognitive Test Battery, *VOMS* Vestibular/Ocular Motor Screening, *SCAT2* Sport Concussion Assessment Tool Version 2, *SCAT3* Sport Concussion Assessment Tool Version 3, *SCAT5* Sport Concussion Assessment Tool Version 5, *ImPACT* Immediate Post-Concussion and Cognitive Testing, *SAC* standardized assessment of concussion, *PCSS* Post-Concussion Symptom Scale, *VA* Video Assessment, *BESS* Balance Error Scoring System, *mBESS* modified Balance Error Scoring System, *MotCoTe* Motor Cognitive Test battery, *PCS* prospective cohort study, *RCS* retrospective cohort study

### Characteristics of included studies

Twenty-six studies (Table 2) met the review inclusion criteria and reported interpretable data on side-line assessments for the diagnosis of concussion. Characteristics of the included studies are summarised in Table 2. Studies consisted of prospective (n = 12), pilot (n = 2), cross sectional (n = 2), prospective observational (n = 1), prospective longitudinal (n = 1), cohort (n = 1), observational (n = 1), descriptive (n = 1), prospective cross sectional (n = 1), pilot case study (n = 1), case control (n = 1) study and retrospective (n = 2). Gender was reported in 24 of the 26 studies with a total population of 7127 participants (5449 males and 1678 females). Twenty studies examined baseline to post-concussion testing. The remaining six studies examined post-concussion exclusively. All studies assessed concussion within the acute diagnosis phase (≤ 7 days). A definition of acute concussion diagnosis was defined in the included studies within the first 3 to 5 days post-concussion [21], ≤ 48 h after concussion for SCAT3 [22], 24–48 h for BESS, SAC testing, subacute measures of neurocognitive impairment (i.e. ImPACT at 5–7 days and 10–14 days post-injury) [23], 24 h [24] and 72 h post-injury [25]. Included studies were conducted between 2015 to 2020 in a range of countries, including the USA, United Kingdom, Finland, Australia, Canada, South Africa and New Zealand.

### Test categories

Included studies (Table 2) used a battery of tests in the diagnosis of side-line concussion (i.e. ‘side-line’ refers to pitch-side, rink side, changing rooms, or an assessment area immediately available that is not a clinical or hospital setting), that fell into three main testing categories (i) cognitive, (ii) observational and (iii) visual. Fifty-six percent of studies employed cognitive tests, 8% observational, a further 8% visual and 28% used a combination of the three test categories. Nineteen studies used one test category, six studies used two test categories and one study reported the use of tests within all three categories (Additional file 1).

Cognitive tests were most commonly employed (n = 16) with a combination of cognitive and observational tests used in a further four studies [23, 26–28]. Two studies used a combination of cognitive and visual [29, 30]. Of the 16 studies that recorded the use of cognitive tests, seven studies used one cognitive test, six studies used two cognitive tests and one study used three cognitive tests. Of the studies that recorded the use of observational testing only, one study used one test while the second study used three testing methods. The two studies that used visual tests exclusively, one test method per study were recorded. The remaining seven studies [23, 26–28] used a combination of cognitive and observational test methods, cognitive and visual test methods [29, 30] or all three test categories [25].

### Tests and screening methods used

A total of 20 different concussion diagnosis tests employed across all studies, differing in frequency. Tests included King–Devick (KD; 10 studies), Sports Concussion Assessment Tool (SCAT) version 3 (SCAT3; 9 studies), Balance Error Scoring System (BESS; 5 studies), Standardised Assessment of Concussion (SAC; 5 studies), tandem gait (TG; 3 studies), immediate post-concussion assessment and cognitive testing (ImPACT; 4 studies), video assessment (VA; 1 study), SCAT version 2 (SCAT2; 2 studies) and vestibular and oculomotor screening (VOMS; 2 studies). The following 11 tests were used once within the range of 16 studies (Brief Symptom Inventory-18, Gait Initiation, Mobile Universal Lexicon Evaluation System, Pitch-Side Concussion Assessment, BTrackS Balance Test, Post-Concussion Symptom Scale, Pitch-Side Concussion Assessment (version 2), CogSport, modified Balance Error Scoring System, Motor Cognitive Test battery, SCAT5). SCAT3 was used on its own in three studies [21, 31, 32]. Three studies used SCAT3 and KD testing in combination with each other [33–35]. Three studies used KD, individually [29, 36, 37]. One study used TG testing along with a combination of cognitive tests [26]. VOMS was used along with a set of cognitive/cognitive and observational tests [25, 29]. One study assessed the diagnostic accuracy of their own developed HIA test which incorporated elements of the SCAT3 [38]

### Administration of tests

Tests were administered by medical and non-medical personnel. Eighty-eight percent of participants were tested for concussion by doctors, clinicians, orthopaedic support, neurologists, or the assistance of certified athletic trainers or physiotherapists. Twelve percent of studies used team members, trained volunteers or the study research coordinator to conduct side-line concussion testing for scientific purposes only.

### Level of sport

A range of different sports and levels of participation were reported in the included studies. For example, sports included rugby (union and league), American Football, ice hockey, baseball, soccer, Australian rules football, basketball, volleyball and wrestling. The sport activities can be divided into two different levels of participation;(1) professional/elite, semi-professional and (2) amateur/club, community level sports, 56% of participants fell into the professional/elite or semi-professional levels of sport, 36% were categorised as amateur/club or community sports. Another 4% were classified as a mixed level cohort of participations who were professional/elite, semi professional and amateur level athletes within a study. The remaining 4% did not state the level of sports played by those participants, however it is important to note the study did not report the level of sports played and testing was carried out within multidisciplinary concussion centres.

### Most significant symptoms

Two studies employing the SCAT3 reported symptom frequency. Fatigue or low energy, along with neck pain [31] and pressure in head, headache and “don’t feel right” [32] were reported as the most common post-injury symptoms. One study [39] observed “slow to get up” a total of 2240 times on 223 different occasions. Signs of “clutching” were reported 212 times during concussion assessment (58.7%). Other concussion diagnosis signs reported within this study included unresponsiveness (n = 52), gait ataxia (n = 102), vacant stare (n = 98), and a post-impact seizure (n = 4). The study by Fuller et al. [38] asserted that self-reported symptoms and observed clinical signs were the strongest predictors of diagnosed concussion, while conversely immediate memory, tandem gait and Maddock’s questions were weak and not significant predictors of concussion.

### Test scores and gender

Two of the 26 studies examined comparison of test scores between females and males. Results were significantly different when compared to each other. Both of these studies used SCAT3 as part [33] or all [21] of the testing protocols. It was clear the results were not similar when compared to each other and further investigation is warranted. For example, Downey et al. [21] reported that while using SCAT3, male participants reported significantly more symptoms (*p* = 0.012), of greater severity (*p* = 0.025); and performed significantly worse on the SAC compared to females (*p* = 0.012). While the study by Harrold et al. [33] reported that using SCAT3, women reported more total symptoms (*p* = 0.001, linear regression, accounting for age) and had higher symptom severity scores (*p* = 0.006).

## Discussion

This systematic literature review is an analysis of current side-line assessments for the diagnosis of concussion in adults participating in professional, semi-professional and amateur sports. A definition of ‘pitch-side’ in this review included side-line, rink side, changing rooms, or an assessment area immediately available that is not a clinical or hospital setting. The focus for this review was from 2015 onwards to align with the most recent consensus statement of concussion in sport (October 2016), as the authors deemed any study carried out prior to that year (2015) would be outdated. The main findings showed studies overall were of moderate to good quality [20] and a variety of cognitive, observational and visual tests were utilised pitch-side by mostly medical and allied health personnel (e.g. physical therapists) to assess acute concussion in adults. However, the review also found that the majority of studies have investigated mostly professional/elite or semi-professional cohorts, aligning with these studies likely having access to medical and allied health staff to undertake these assessments. Conversely the limitation in non-medically trained researchers operating assessments is likely to be contributing to the paucity of studies being undertaken at non-elite/club levels of participation where research is urgently required.

Key findings from the qualitative review showed a large range of different assessments used to quantify concussion. Interestingly, we found that less than half of the studies employed the SCAT assessment (either SCAT2, 3 or 5 for this systematic review). We found this observation surprising given that it has been suggested that the SCAT is the most widely accepted and deployable sport concussion assessment and screening tool currently available [15]. We appreciate that the use of SCAT in research is not a mandatory requirement, and that the objective of studies would be to test the efficacy of other modalities and cohorts, such as in a laboratory environment. However, in field studies such as those included in this review, to reduce disparity in findings, the SCAT assessment should be used consistently across future studies.

The review also found that ~ 25% of studies used a multi-modal approach to assess concussion by combining two or more testing batteries. While it may be argued that the SCAT does incorporate a multi-modal approach [1], there are elements that the SCAT assessment does not measure, such as the oculo-motor system [15]. Indeed, a previous study where the SCAT and an oculomotor test such as the King–Devick (KD) test have been implemented together, results showed a 100% sensitivity in diagnosing athletes suspected of concussion [40]. For clinically focused studies and application to clinical-practice, future work should incorporate two or more rapid and non-invasive pitch-side assessments for the diagnosis of concussion to reduce the risk of false-positives or false-negative diagnoses occurring, which may affect follow up results. This has been recently suggested in a systematic review by Harris et al. [41].

An ongoing concern, particularly for concussion in non-elite/community club-based sports, is the paucity of suitably qualified people who are allowed to administer the SCAT HIA. This is reflected in the current review where the majority of studies used SCAT at professional/elite levels of sport, where access to a medical practitioner was possible. As the consensus statement strictly asserts that only a medical practitioner can administer the SCAT as part of the clinical diagnosis [1], this may limit opportunities for suitably qualified scientists who are technically proficient at the SCAT but cannot provide a result, limiting research. Conversely other assessments such as the VOMS can be delivered by allied health professionals and the KD can be delivered by anyone, increasing their potential usage in research, but due to aforementioned limitations this would be without the use of the SCAT (despite assertions that the SCAT is the most widely agreed upon assessment tool for concussion) [15]. As studies have argued that when used in isolation assessments such as the KD or VOMS may not be sensitive enough alone to detect concussion [37, 42], we suggest from this systematic review that the SCAT should be incorporated with other testing modalities for data collection purposes, with any clinical diagnosis made by the associated team doctor outside of the study scope.

Interestingly, despite consensus on the use of video identification of concussion [43], only one study in this systematic review utilised video. A reason for this was because the video was used for post-event confirmation rather than used for confirmation of concussion at time of incident. With network media covering professional/elite events, video is easily accessible at matches. However, although multiple and multi-angled cameras are not available at amateur/community club levels, many sub-elite competitions will now incorporate a fixed camera supplied by the clubs themselves or via the local league for streaming or replay on the league’s webpage. While not optimal in terms of clarity, it may assist the detection of concussion and we suggest that future studies involve video to confirm concussion events to improve study quality. Similarly, we found no studies employing impact sensors in a surveillance capacity. The use of impact sensors (attachments behind the ear or embedded within a gumshield/mouth guard) will assist video confirmation of suspected concussion events, however with no studies to support this hypothesis, we conclude no studies using impact sensors were eligible for inclusion in this systematic review.

Limitations of this review include research involving lab-based clinical assessments. However, the focus of this review was on published research that could assist at the lower levels of sport in community or amateur settings (i.e. in settings where the presence of medical professionals’ pitch-side may be limited or indeed non-existent). Another limitation we acknowledge is that the laws of each individual sporting code may not allow for head injury assessments during the game: this may influence timing of assessments and the ability to 'Recognise and Remove'. In these instances, the general approach would be to remove the participants from the sports activity where there is any indication that a concussion has occurred. These initial sideline tests may be substantiated at a later time using advanced diagnostic techniques by a medical professional or video analysis. The studies included in this review utilised well established testing methods which offered some form of side-line tests for suspected concussions and have highlighted the necessity for a multimodal concussion assessment tool for the initial identification and assessment of concussion. This review highlighted the need for multi-modality concussion testing and that there is a clear disparity in research focusing on professional/elite levels and the lack of studies in amateur/club levels.

## Conclusions

Recognising suspected concussion in sports participants is most effectively realized by using multimodal test protocols that are guided (via primary or secondary confirmation) by medical experts. Based on this review, the KD and SCAT (versions 2 and 3) appear to be the most commonly used tools for the primary assessment of concussion currently. Using additional tests such as VOMS from an observational perspective and balance testing such as BESS show promise in conjunction with cognitive testing. The addition of concurrent video review could potentially offer a promising approach to improve identification and evaluation of significant head impact events, and a multi-modality-based concussion evaluation process appears to be important to detect delayed-onset SRC, however current evidence does not support the use of impact sensor systems for real-time concussion screening. As shown in a recent systematic review [41] there is an urgent need to conduct research, using multi-modality assessment methods, but focusing on non-elite levels where concussion injuries occur regularly but a lack of resources and education preclude effective assessment and management.

## Supplementary Information

**Additional file 1.** Categorisation of test type and the tests included in each.

**Additional file 2.** PRISMA checklist.

## Data Availability

Available upon request (please contact Dr Lisa Ryan, lisa.ryan@gmit.ie).

## Abbreviations

KD: King Devick test
GI: Gait Initiation
SOT: Sensory Organization
TG: Tandem Gait
GT: Gait Termination
MULES: Mobile Universal Lexicon Evaluation Systems
PSCA: Pitch-Side Concussion Assessment Version 1
PSCA2: Pitch-Side Concussion Assessment Version 2
MotCoTe: Motor Cognitive Test Battery
VOMS: Vestibular/Ocular Motor Screening
SCAT2: Sport Concussion Assessment Tool Version 2
SCAT3: Sport Concussion Assessment Tool Version 3
SCAT5: Sport Concussion Assessment Tool Version 5
ImPACT: Immediate Post-Concussion and Cognitive Testing
SAC: Standardized assessment of concussion
PCSS: Post-Concussion Symptom Scale
VA: Video Assessment
BESS: Balance Error Scoring System
mBESS: Modified Balance Error Scoring System
MotCoTe: Motor Cognitive Test battery
PCS: Prospective cohort study
RCS: Retrospective cohort study
