# Supplementary material for: An assessment of current concussion identification and diagnosis methods in sports settings: a systematic review
Source: BMC Sports Sci Med Rehabil. 2022 Jul 10;14:125. doi: 10.1186/s13102-022-00514-1 (PMC9275058; doi:10.1186/s13102-022-00514-1)
Supplement: Supplementary file 1 — Additional file 1. Categorisation of test type and the tests included in each. [file 13102_2022_514_MOESM1_ESM.docx]

**Additional File 1**

**Categorisation of test type and the tests included in each.**

| **Test Type** | **Test Used** |
| --- | --- |
| **Cognitive** | **KD:** King-Devick  **SCAT 5:** Sports Concussion Assessment Tool - version 5  **SCAT 3:** SCAT - version 3  **SCAT 2:** SCAT - version 2  **SCA:** Standardised Assessment of Concussion  **ImPACT:** Immediate Post-concussion Assessment Cognitive Testing  **BSI:** Brief Symptom Inventory-18  **PSCA:** Pitch Side Concussion Assessment  **PSCA-2:** Pitch Side Concussion Assessment (version 2)  **PCSS:** Post-Concussion Symptom Scale  **CogSport** (Cognitive Function Test)  **MCTB:** Motor Cognitive Test battery |
| **Observational** | **BESS:** Balance Error Scoring System  **mBESS:** modified Balance Error Scoring System  **TG:** Tandem Gait  **BTBT:** BTrackS Balance Test  **VA:** Video Assessment |
| **Visual** | **VOMS:** Vestibular/Ocular Motor Screening  **MULES:** Mobile Universal Lexicon Evaluation System  **GI:** Gait Initiation |
